# The association among teacher-student relationship, subjective well-being, and academic achievement: Evidence from Chinese fourth graders and eighth graders

**DOI:** 10.3389/fpsyg.2023.1097094

**Published:** 2023-01-26

**Authors:** Da Zhou, Shuting Liu, Hao Zhou, Jian Liu, Yue Ma

**Affiliations:** ^1^Faculty of Education, Northeast Normal University, Changchun, China; ^2^Collaborative Innovation Center of Assessment for Basic Education Quality, Beijing Normal University, Beijing, China; ^3^School of Education, Shanghai Jiao Tong University, Shanghai, China

**Keywords:** teacher-student relationship, subjective well-being, academic achievement, grade levels, Chinese students

## Abstract

This study compared the teacher-student relationship, subjective well-being, and academic achievement between fourth graders and eighth graders, and examined whether and how subjective well-being played a mediating role concerning teacher-student relationship and academic achievement across the two grades. The participants included 19,845 fourth graders and 11,691 eighth graders from a city in central China. The findings indicated that (1) compared with eighth graders, fourth graders reported significantly higher mean scores in the teacher-student relationship, subjective well-being, and academic achievements; (2) a positive teacher-student relationship can promote students’ academic achievement both directly and indirectly through subjective well-being across the two groups; (3) The mediating effect of subjective well-being accounted for 42.8% of the total effects between teacher-student relationship and academic achievement for fourth graders, which was higher than that for eighth graders (22.7%). Limitations and future directions are elaborated.

## Introduction

Establishing and sustaining high-quality interpersonal relationships not only promotes people’s physical and mental health across different developmental stages but also is a key aspect of achieving a variety of positive outcomes (Poling et al., 2022). In the classroom context, the relationships between teachers and students built may have a positive or negative impact on students’ emotions, behavior, and cognition (Cornelius-White, 2007; Barile et al., 2012; Roorda et al., 2017). Specifically, the teacher-student relationship is a two-way interpersonal association that takes place in proximal (e.g., proactive interactions) and distal systems (e.g., the classroom environment; Zhou et al., 2020). When a supportive teacher-student relationship is established, students are more willing to express their ideas and collaborate with their peers, which stimulates students’ motivation, helps them experience a sense of well-being, and, in turn, achieves academic success (Barile et al., 2012; Li, 2021). Further, when such students are faced with challenging tasks or setbacks, they are more likely to persevere, especially with the encouragement, feedback, and support of their teachers (Koca, 2016). However, a negative teacher-student relationship can hinder students’ autonomy, subjective well-being, and the satisfaction of related needs, and, in turn, harm their academic performance (Spilt et al., 2011; Lei et al., 2022). It is therefore vital to focus on the effects of the teacher-student relationship in the classroom context.

The affective quality of the teacher-student relationship is an important predictor of students’ emotions. Subjective well-being is one of the more important student emotional factors (Roorda et al., 2011). Subjective well-being is popularly referred to as happiness or satisfaction, including both reflective cognitive judgment, such as life satisfaction, and emotional responses to ongoing life in terms of positive and pleasant emotions versus unpleasant and negative emotions (Diener et al., 2018). Research has found that about 60 to 70% of subjective well-being was attributable to environmental effects (Diener et al., 2018). Further, in a survey of 123 countries, researchers have also found that basic and psychological needs are closely linked to students’ well-being (Tay and Diener, 2011). In school education, a supportive and secure teacher-student relationship precisely provides a good basis for students’ psychological satisfaction (Zhou et al., 2020), so from the perspective of attachment theory (Baker et al., 2008), a teacher-student relationship may play an important role in developing students’ well-being. Additionally, self-determination theory also indicated how social and contextual factors facilitate or undermine people’s sense of volition and initiative, and their well-being (Ryan and Deci, 2017; Amholt et al., 2020). Although some theories have suggested the link between the teacher-student relationship and students’ subjective well-being, some empirical evidence is not still enough. Thus, it is also important to examine the influence of the teacher-student relationship on students’ subjective well-being based on large-scale survey data of different groups, and thus help us deeper understand the links between them.

In recent years, a new focus on subjective well-being is exploring its outcomes. According to the theory of broaden-and-build of positive emotions (Fredrickson, 2001), positive emotions broaden a person’s mindset and, in educational contexts, increase attention to learning, which, in turn, develops successful academic outcomes. In contrast, negative emotions (e.g., sadness and anxiety) are thought to hamper a person’s cognition development, thereby hindering students’ learning and leading to failure in academic achievement (Bortes et al., 2021). Some empirical results also supported the theoretical explanations. For example, King et al. (2015) found that students with higher levels of subjective well-being tend to be more engaged in learning. Amholt et al. (2020) conducted a systematic review of the links between subjective well-being and academic achievement and found that there are inconsistent research results regarding the relationships between them. A possible explanation for the conflicting results in prior studies may be related to students’ grade levels (Romero et al., 2014; Bücker et al., 2018; Amholt et al., 2020). When children increase their grade levels, they will spend more time on their homework and on trying to get better academic performance (Ai, 2015; Ma, 2015). Specifically, pupils have higher positive emotions (e.g., subjective well-being, enjoyment) when starting school education, but as they grow older the positive emotions decrease (Pekrun, 2006). The change in emotions could lead to different developmental trajectories of well-being and academic performance over time. In addition, as children grow and mature mentally, the role of the teacher-student relationship will also change, which will have a different impact on students’ outcomes (Hamre and Pianta, 2001). Thus, grade levels may be an important factor for series associations among the teacher-student relationship, subjective well-being, and academic performance.

In general, this study will explore the association among teacher-student relationship, subjective well-being, and academic achievement across different grade levels. Theoretically, this study may help deepen the understanding of the psychological mechanisms of the three variables and explain why prior results are inconsistent, and practically, it may provide insights for educators to design grade-appropriate interventions to enhance students’ subjective well-being and academic achievement.

## Literature review

### Teacher-student relationship and academic performance

The teacher-student relationship is usually regarded as one of the most important interpersonal relationships in the classroom context. Especially, the teacher-student relationship is a dyadic social process that comprises ongoing interactions between teachers and students in classrooms (Robinson, 2022). Teachers and students go through a process of meeting one another, exchanging information, and adjusting and developing expectations, so the teacher-student relationship was regarded as teachers’ and students’ interactions over time, affect each other and aggregate perceptions of one another (Frymier and Houser, 2000; Robinson, 2022). In the last 20 years, the effect of the teacher-student relationship on students’ academic performance has been supported by relevant theoretical and empirical studies (Cornelius-White, 2007; Zhou et al., 2020).

John Bowlby’s attachment theory (1981) is one of the most influential perspectives on exploring the role of the teacher-student relationship, especially for pupils (Zhou et al., 2021). In the last two decades, attachment theory has become one of the most important perspectives for understanding the process of affect regulation (Mikulincer et al., 2003). Especially, when infants interact with significant others who are available in times of need, this interrelationship facilitates the optimal functioning of the system and promotes the formation of a sense of attachment security, so the attachment theory usually thought that an infant who views his or her parents as a secure base can explore and learn with fewer worries and less distraction (Mikulincer and Shaver, 2010). When attachment theory was transferred to pupils, attachment theory played a similar role in supporting the effect of the teacher-student relationship on students’ academic achievement in the classroom context (Cornelius-White, 2007; Zhou et al., 2020; Poling et al., 2022). A supportive teacher-student relationship based on the attachment theory can provide a secure foundation for pupils to enjoy learning, develop new skills, autonomously pursue new goals, and realize their potential and aspirations, which will promote students’ progress in academic performance (Mikulincer and Shaver, 2010; Zhou et al., 2020, 2021). Some studies have found that a warm teacher-student relationship has a significant influence on pupils’ academic performance, such as mathematics, problem-solving, and reading performance (Hughes et al., 2008; Roorda et al., 2011; Zhou et al., 2020, 2021). A meta-analysis also showed that there exists a positive link between the teacher-student relationship and pupils’ reading, mathematics, and science performance (Cornelius-White, 2007).

In addition, self-determination theory is also one of the most theories for exploring the effects of the teacher-student relationship, especially for middle school students (Davis, 2006). Self-determination theory assumes that individuals are motivated and have a desire for self-realization and self-growth (Ryan and Deci, 2011), so three basic psychological needs (e.g., autonomy, competence, and relatedness) are necessary (Bakadorova and Raufelder, 2018). Specifically, central to self-determination theory is autonomy motivation (Gagne and Deci, 2005). Autonomy usually involves acting with a sense of volition and having the experience of choice. For example, when people engaged in an activity because they found it interesting, they are doing the activity wholly volitionally (Gagne and Deci, 2005; Ryan and Deci, 2006; Hu and Zhang, 2017). In the classroom context, a warm relationship was precisely a prerequisite for effectively engaging in activities, so autonomy motivation to self-determination theory provided a theoretical rationale for supporting the role of the teacher-student relationship in this study. In school education, the relationships between teachers and students constitute the classroom context. A warm relationship will foster students’ relatedness and autonomy. For example, students are willing to express autonomously their ideas and explore methods to solve problems in the classroom, and teachers also can motivate students to learn more, engage with more, understand better, and adapt instruction to address students’ needs, thereby achieving students’ better academic performance (Lei et al., 2022). A study investigating 523 grade 8 students found that eighth graders who perceived the positive teacher-student relationship expressed more positive school-related affect and were able to develop their academic competence from the self-determination theory’s perspective (Chong et al., 2010). In addition, a meta-analysis conducted by Quin (2017) analyzed 46 published studies focusing on the sample of middle school students and high school students and found that the teacher-student relationship still has a significant impact on student’s academic performance even after controlling for individual-, family-, school-, and teacher-level factors.

Theoretically, both the attachment theory and self-determination theory supported the role of the teacher-student relationship on students’ academic performance. However, there is still a lack of empirical evidence in comparing the effects of different theories. Hence, this study was to empirically examine those relationships among elementary school students and middle school students, which will further deepen the current studies.

### The mediating role of subjective well-being

Subjective well-being is a broad category of phenomena that includes people’s emotional responses and global judgments of life satisfaction (Diener and Ryan, 2009; Luhmann, 2017). Specifically, moods and emotions, which together are labeled affect, represent people’s ongoing evaluations of the events that occur in their lives. Diener and Emmons (1984) found that pleasant (e.g., joy, happiness, contentment, and pride) and unpleasant effects (e.g., sadness, anger, stress, depression, and envy) formed two key aspects of moods and emotions and became increasingly separate as the timeframe increased. In addition to paying attention to emotional responses, researchers of subjective well-being also are interested in the cognitive evaluation of life satisfaction, including the desire to change a life, satisfaction with current life, past life, future life, and significant others’ views of one’s life (Diener and Ryan, 2009; Diener et al., 2018). Apart from emotional responses and life satisfaction, domain satisfaction should be considered as one aspect of subjective well-being such as work satisfaction, family satisfaction, financial health, and so on (Diener et al., 1999). Because subjective well-being is not a unitary phenomenon, researchers must study separately each of the components. Therefore, emotional responses were considered a key aspect of subjective well-being in school education and were measured independently in this study.

In previous studies, some researchers have explored the factors that influence subjective well-being, outcomes of subjective well-being, theoretical processes associated with subjective well-being, and so on (Diener et al., 2018). Specifically, in the factors that influence subjective well-being, Siedlecki et al. (2014) explored the effects of social support on subjective well-being and found that various forms of assistance supplied by family members, friends, neighbors, and others can affect people’s life satisfaction. In school education, Bakadorova and Raufelder (2018) conducted a longitudinal study to examine the association between the teacher-student relationship and middle school students’ subjective well-being and found that there exists a positive link between the teacher-student relationship and students’ subjective well-being. In their studies, teacher-student relationship and subjective well-being were mainly supported by attachment theory and self-determination theory, meaning that students’ perception of a warm teacher-student relationship leads to a safer atmosphere where students are more motivated to express themselves and thus feel fulfilled (Siedlecki et al., 2014; Bakadorova and Raufelder, 2018; Diener et al., 2018). Further, Van Petegem et al. (2007) also focus on the effect of interpersonal relationships on students’ well-being and indicated that a positive classroom climate that was created by teachers and students can contribute to a higher sense of well-being for students learning in technical and vocational secondary schools. However, previous studies just focused more on the samples of undergraduates, middle school students, and elementary school students separately instead of simultaneously paying attention to the difference across grade levels.

In terms of the outcomes of subjective well-being, Ng et al. (2015) conducted a short-term longitudinal study with 821 middle school students to determine the effect of subjective well-being on academic achievement and the results showed that subjective well-being as a process variable positively affects academic achievement as an outcome variable. Bücker et al. (2018) conducted a meta-analysis focusing on the links between subjective well-being and academic achievement and found that the correlation between academic achievement and subjective well-being was small to medium in magnitude and statistically significant at *r* = 0.164, 95%CI [0.113, 0.216]. Another important systematic review study reported that the positive association between subjective well-being and academic performance was primarily cross-sectional, focused on young children, and used parent or teacher ratings in addition to self-report (Amholt et al., 2020). Furthermore, although some studies addressed the reciprocal effects of academic achievement and subjective well-being (Datu and King, 2018; Bortes et al., 2021), but Amholt et al. (2020) found that subjective well-being is mainly considered a prerequisite for several outcomes including academic achievement. This finding of the effect of subjective well-being on academic achievement was supported mainly by the theory of broaden-and-build of positive emotions (Fredrickson, 2001), meaning that the positive emotions brought about by subjective well-being promote students’ thinking and enhance students’ focus on learning, thus facilitating their progress in academic achievement (Bortes et al., 2021).

Although some studies have explored the factor (e.g., teacher-student relationship) that influences students’ subjective well-being and the outcome (e.g., academic achievement) of subjective well-being in the educational context, these studies did not focus simultaneously on the mediating role of subjective well-being on the between teacher-student relationship and academic achievement. Thus, in this case, this study will further explore the mediating role of subjective well-being in an educational context to provide a new explanation and rich the current studies.

### The role of grade levels

Grade levels play an important role in students’ learning and emotional experience (Dalton, 2012). In elementary school, students’ cognitive development level is immature, and they are more influenced by external context, which makes them more susceptible to changes in their emotional experience and learning motivation that can affect their academic outcomes (Dhuey, 2013; Altermatt, 2016; Wang and Tsai, 2017). However, when students enter secondary school, compared with primary school students, they will be at a more mature cognitive development level, they will have their ideas, and be less susceptible to external influences (e.g., a classroom context created by a terrible teacher-student relationship). Changes in students’ perceptions seemed to weaken the impact of emotional experiences (e.g., subjective well-being, life satisfaction, etc.) on their achievement (Cleary and Chen, 2009; Devlin et al., 2022). In addition, the transition from elementary school to secondary school can be a particularly daunting event for many students, because the focus at elementary school is on a typically supportive, master-based orientation, while at middle school, there is a shift to a performance-focused environment characterized by increased expectations of academic productivity, more intensive and teacher-directed teaching, and a greater focus on normative comparisons and high-stakes outcomes (Cleary and Chen, 2009). Therefore, the links among the teacher-student relationship, subjective well-being, and academic performance may differ for pupils and middle school students.

In prior studies, some studies have explored the impact of grade levels on students’ academic achievement and emotional experience, but most of these studies just focus on the difference among different variables (Dalton, 2012; Dhuey, 2013; Wang and Tsai, 2017). However, further exploring the relationships among different variables are also important across different grade levels, which will provide abundant evidence for researchers and teaching practitioners to develop individualized and adaptive interventions or strategies. Thus, this study will further explore the role of grade levels among different variables for ricing the existing research.

Further, fourth graders in primary school and eighth graders in middle school are selected to represent students at both the primary and secondary levels. The specific reasons for this are mainly the following. First, lower primary school students usually have lower literacy skills and are more cognitively engaged than upper primary school students, while upper primary school students need more time to prepare for the entrance examinations to middle school, so, in comparison, it is more suitable to choose fourth graders in the middle-grade level. Second, at the middle school, seventh graders have just started their middle school life and need to adapt it, while ninth graders need to face the entrance examinations to high school, so the choice of eighth graders is more appropriate as the representation of middle school students. In addition, in previous studies, grade 4 and grade 8 were also usually chosen such as PISA and TIMSS. Thus, grade 4 and grade 8 will be regarded as important representations of elementary school and middle school levels.

### The present study

**Figure 1:**
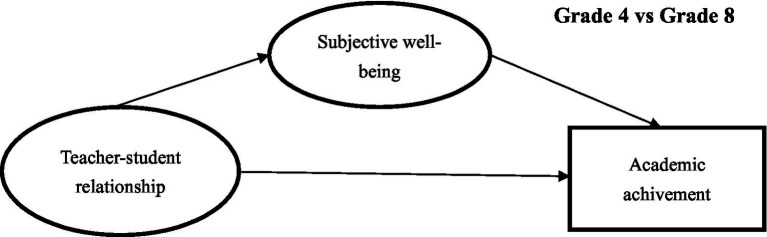
Conceptual framework of the present study.

This study constructed a structural equation modeling (Figure 1) based on the existing studies and related theories. To further provide more abundant empirical evidence to fill in the gaps in the literature, this study proposed four research questions:

Q1: Are there significant differences in the teacher-student relationship (TSR), subjective well-being (SWB), and academic achievement (AA) between fourth graders and eighth graders?

Q2: Is TSR positively associated with AA across fourth graders and eighth graders?

Q3: Does SWB play a significant mediating effect in the relationship between TSR and AA for fourth and eighth grades students?

Q4: If Q3 is supported, does the mediating effect of SWB in the relationship between TSR and AA differ across fourth graders and eighth graders?

## Methods

### Participants

Participants in this study were from a city in central China. Based on the basic requirements of stratified sampling on the sample size (Johnson and Christensen, 2019) and the needs of the local education bureau, we conducted an equal proportion of two stratified random sampling methods to collect data. First, we randomly selected 80% of the schools in each district or county. A total of 304 elementary schools and 164 middle schools were chosen from all 10 districts (or counties). Second, 20% of fourth graders and eighth graders from each school were randomly selected to participate in this study. This resulted in a total of 19,845 Grade 4 students and 11,691 Grade 8 students. Among them, 10,231 (51.6%) were males and 9,613 (48.4%) were females in elementary schools, and 6,064 (51.9%) were males and 5,627 (48.1%) were females in middle schools. The average age of fourth graders was about 10 years, while the average age of eighth graders was 14 years. All students participating in the study were voluntary.

### Measures

#### Covariables information

The effect of socioeconomic status (SES) and gender on academic achievement have been widely validated in educational research (Sirin, 2005; Nowell and Hedges, 2018; Liu et al., 2020; Selvitopu and Kaya, 2021) and therefore need to be controlled as covariates for in this study. Specifically, gender was coded “1” for males and “2” for females. SES was composed of parents’ occupation, education, and family possessions. In this study, principal components analysis was used to calculate a composite value of SES (Organisation for Economic Co-Operation and Development [OECD], 2017).

#### Academic achievement

In this study, we used student mathematics performance as the outcome. There are three main reasons for choosing mathematics achievement. First, compared with other subjects, the mathematics achievement test is the main subject of large-scale achievement tests such as the Program for International Student Assessment (PISA) or the Third International Mathematics and Science Study (TIMSS). Second, this study will compare the academic achievement of elementary and secondary school students, so it is necessary to maintain the consistency of achievement test subjects. Third, mathematics is usually characterized by abstraction and rigor, which makes mathematics learning more difficult for students (Zhou et al., 2020). Therefore, it’s very important to focus on how we should develop students’ mathematics achievement. Based on this, the academic achievement used in this study is a mathematical achievement.

Specifically, the mathematics achievement tests for both grade 4 and grade 8 students have been written by a team of professional experts based on *China’s National Mathematics Curriculum Standards*. For the mathematics achievement test of grade 4 and grade 8, there are 27 items and 25 items, respectively, each comprising the content domains and the cognition domains (read more details of the middle school and elementary school test development in Liu et al., 2019; Zhou et al., 2021, 2022). The mean and standard deviations of the normalized conversion scores of students’ overall scores were 500 and 100, respectively. The internal consistency (Cronbach’s alpha) of the mathematics achievement test was 0.851 (Year 4) and 0.895 (Year 8), which are acceptable because greater than 0.80 (Cortina, 1993).

#### Teacher-student relationship

The teacher-student relationship questionnaire was designed to investigate students’ perceived relationships with their mathematics teachers. Specifically, this questionnaire was adopted from the 2012 Program for International Student Assessment. It included five items, which scored 5 points from “strongly disagree (1)” to “strongly agree (5).” When students scored higher, meaning that they perceived better relationships with their teachers. The model fit indices of the questionnaire were accepted (Hu and Bentler, 1999), and the internal consistency reliability of the questionnaire as estimated by Cronbach’s alpha was also reasonable (see Table 1).

**Table 1:** Validity and reliability of teacher-student relationship (TSR) and subjective well-being (SWB) questionnaire.

Questionnaire	Groups	CFI	TLI	RMSEA	SRMR	*x* ^2^	*df*	*α*
TSR	Grade 4	0.984	0.969	0.059	0.017	1180.218	5	0.913
	Grade 8	0.989	0.977	0.057	0.012	671.568	5	0.926
SWB	Grade 4	0.969	0.956	0.073	0.018	6818.964	20	0.973
	Grade 8	0.953	0.934	0.082	0.023	5433.603	20	0.962

#### Subjective well-being

We used the Index of Well-Being (IWB; Campbell, 1976) to measure students’ subjective well-being (SWB). The IWB is a nine-item scale designed to assess students’ life satisfaction (the first item) and general effects (the rest items). For considering the construct validity of the questionnaire, we used the rest items as the test tool of SWB. This scale is self-reported, in which students are asked to rate to which extent they agreed with the statements on a seven-point Likert scale (1 = not a lot to 7 = a lot). Higher total scores indicate higher levels of SWB. The IWB scale has been adopted in China with satisfactory validity and reliability (e.g., Zhang et al., 2004). The model fit indices of the scale were acceptable, and Cronbach’s alpha coefficient was considered acceptable (see Table 1).

### Procedures

To ensure the process validity of this study, we followed a scientific data collection process. First, students were invited to complete a mathematics achievement test within 80 min. Then, they were invited to take an online survey, including teacher-student relationship and subjective well-being questionnaires within 15 min. In the process of collecting data, all students received the same instructions. Third, for scoring students’ performance on the test, we recruited a group of postgraduate students who majored in mathematics education, and they were trained to grade students’ achievement tests with the given scoring criteria (Zhou et al., 2022). When they were achieving a high scoring consistency (90%), they were allowed to formally code with the test. Finally, we also employed a company to independently enter all survey scores on tests and questionnaires for further data analysis.

### Data analysis

The study mainly follows four procedures to analyze data. First, a two-factor confirmatory factor analysis (CFA) was used to examine the construct validity of instruments of the teacher-student relationship and subjective well-being at Grade 4 and Grade 8, respectively (McInerney et al., 2012).

Second, descriptive statistics analysis and correlation analysis were used to examine the distributions and differences of all the variables used in this study, and the associations among covariables and other variables in Grade 4 and Grade 8, respectively. Additionally, we also calculated effect sizes to reflect the magnitude of the absolute effects, ignoring the confounding effect of sample size on the significance between variables (Funder and Ozer, 2019; Zhou et al., 2022).

Third, we conducted structural equation modeling (SEM) analysis to explore the structural relationships among teacher-student relationships, subjective well-being, and academic achievement (Wang and Wang, 2020). We preferred SEM analysis over path analysis as it considered the measurement errors by using a latent analysis approach.

Finally, we calculated bias-corrected bootstrap tests with a 95% confidence interval (Gootzeit and Markon, 2011) to examine whether the indirect effects were significant, and then we compared the percentage of the indirect effects in each group (grade 4 vs. grade 8), respectively. It is worth noting that some researchers used Multi-group SEM to explore the significant differences between different paths across two groups. However, the purpose of this study just focused on the proportion of indirect effects in the total effect, so we did not need to use this method instead of directly calculating the percentage of indirect effects.

## Results

### Confirmatory factor analysis

To examine the measurement structure of the questionnaire in grade 4 and grade 8, this study conducted CFA on the two latent variables, i.e., the teacher-student relationship and subjective well-being. The results of CFA showed that the two-factor structure presented a good fit for both grades: in Grade 4, χ^2^ (64, 19,844) = 8290.477, *p* < 0.001, RMSEA = 0.080, CFI = 0.972, TLI = 0.966, SRMR = 0.019, and in Grade 8, χ^2^ (64, 11,682) = 6427.688, *p* < 0.001, RMSEA = 0.092, CFI = 0.964, TLI = 0.956, SRMR = 0.023. In addition, all latent variables had high factor loadings (see Table 2). These results revealed that the two instruments in this study had good construct validity for both students of grade 4 and grade 8 (McInerney et al., 2012). That means the two constructs were reasonable for further analysis.

**Table 2:** Standardized factor loadings of two variables in grade 4 and grade 8.

	Items	Grade 4	Grade 8
Teacher-student relationship (TSR)	tsr1	0.833	0.869
	tsr2	0.906	0.929
	tsr3	0.869	0.922
	tsr4	0.851	0.893
	tsr5	0.746	0.835
Subjective well-being (SWB)	swb1	0.842	0.833
	swb2	0.904	0.890
	swb3	0.920	0.908
	swb4	0.894	0.837
	swb5	0.943	0.919
	swb6	0.943	0.932
	swb7	0.932	0.927
	swb8	0.893	0.861

All loadings are statistically significant (*p* < 0.001).

### Descriptive statistics and correlations analysis

To explore the differences in SES, teacher-student relationship, subjective well-being, and academic achievement across grade 4 and grade 8, independent T-tests were used. Table 3 presents the T-test’s results. The results showed that the SES, teacher-student relationship, subjective well-being, and academic achievement of fourth graders were significantly higher than those of eighth graders. No significant gender difference was found in the two grades. Considering the large sample size, this study also further calculated the Cohen’s *d* value as an important indicator to describe how large the difference of all variables between the two grades. Cohen’s d results further indicated there existed four medium effects of SES (*d* = 0.28), teacher-student relationship (*d* = 0.39), subjective well-being (*d* = 0.33), and academic achievement (*d* = 0.20) across the two grades. To summarize, the fourth graders performed stronger in academic achievement and better in emotional variables (see Table 3).

**Table 3:** Summary of means, standard deviations, a one-way between-group Anova, and Cohen’s d in students in grade 4 (*n* = 19,844) and grade 8 (*n* = 11,682).

	Grade 4		Grade 8		T-test		*Cohen’s d value*
	*M*	*SD*	*M*	*SD*	*t*	*df*	
Gender	1.49	0.5	1.49	0.5	0.46	31,524	0.00
SES	0.24	0.67	0.07	0.71	20.22***	21,038	0.28
TSR	4.22	0.91	3.90	0.90	29.90***	24,054	0.39
SWB	5.32	2.02	4.74	1.77	26.50***	26,282	0.33
AA	555.61	83.18	540.72	84.84	15.14***	24,072	0.20

SES, Socioeconomics Status; TSR, Teacher-student Relationship; SWB, Subjective Well-being; AA, Academic achievement. ***p < 0.001.

To determine the extent to which all study variables correlated with each other, the Pearson correlation analysis was used. The results indicated that SES and gender were associated significantly with all other variables for the fourth and eighth graders. Therefore, for conducting further analysis, both SES and gender need to be controlled in the two grades. In addition, the results also showed that the teacher-student relationship was positively correlated with academic achievement for the fourth graders (*r* = 0.353, *p* < 0.01) and the eighth graders (*r* = 0.202, *p* < 0.01), which confirmed research question 2 (see Table 4).

**Table 4:** Summary of correlation coefficients in grade 4 and grade 8.

	Gender	SES	TSR	SWB	MA
Gender	1	0.022**	0.022**	0.031**	−0.049**
SES	−0.001	1	0.132**	0.143**	0.353**
TSRs	−0.023*	0.152**	1	0.227**	0.108**
SWB	0.023*	0.109**	0.295**	1	0.175**
MA	−0.043**	0.352**	0.202**	0.144**	1

Descriptive results presented Grade 4 above the diagonal (*n* = 19,844), and Grade 8 below the diagonal (*n* = 11,682). *p < 0.05; **p < 0.01.

### Structural equation modeling analysis

SEM was used to examine the relationships among the teacher-student relationship, subjective well-being, and academic achievement. This study proposed that the positive relationship between the teacher-student relationship and academic achievement was mediated by subjective well-being in the two grade levels. Results suggested that the data fit reasonably the proposed models. Table 5 presents the fit indices of both models.

**Table 5:** Fit indices of two models.

	*x* ^2^	*df*	CFI	TLI	RMSEA	SRMR
Grade 4	9165.396	101	0.970	0.964	0.067	0.041
Grade 8	7001.071	101	0.961	0.954	0.076	0.037

CFI, comparative fit index; RMSEA, root mean square error of approximation; SRMR, standardized root means square residual.

**Figure 2:**
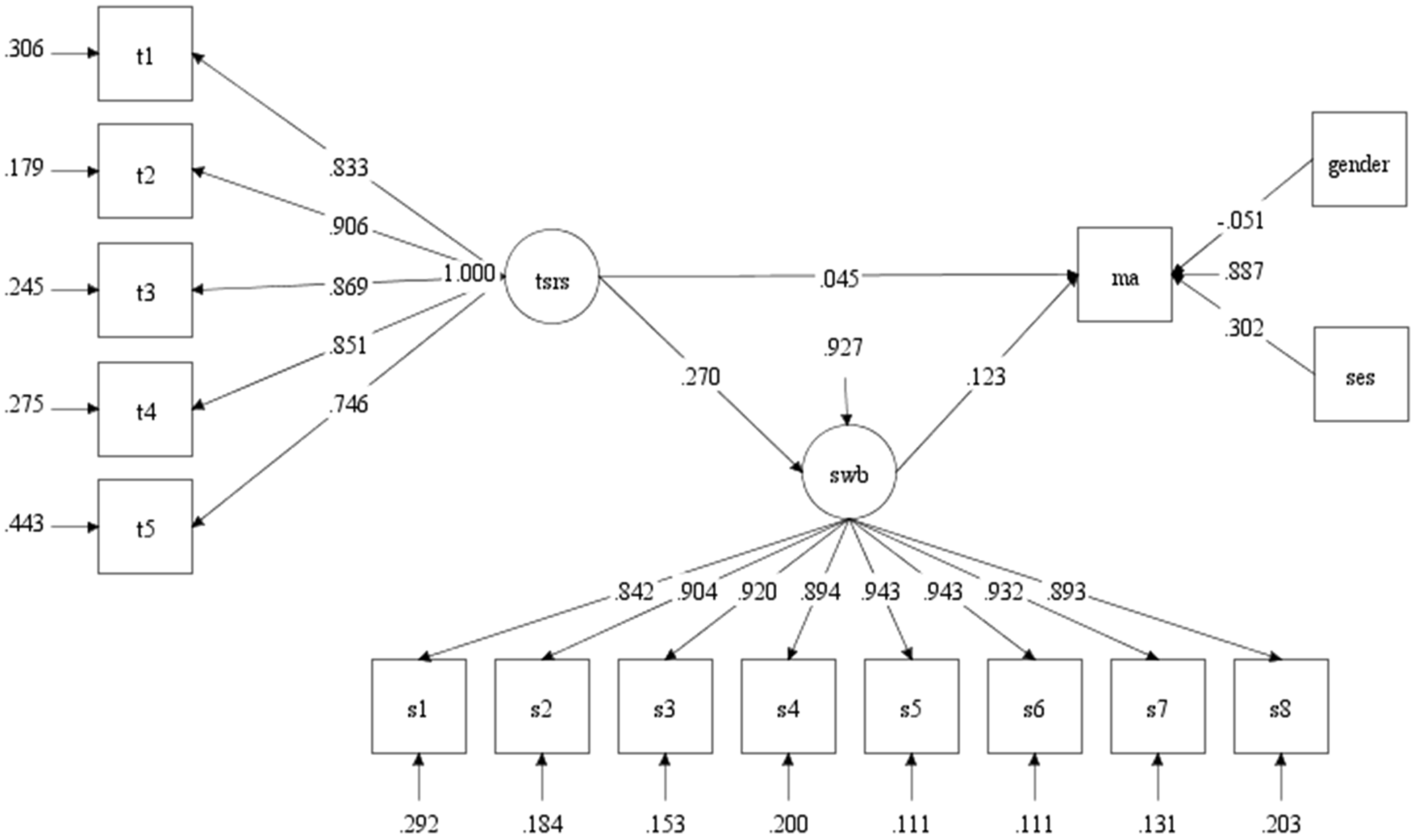
The results of SEM analysis in grade 4. TSR , Teacher-student relationship; SWB,  Subjective well-being; MA,  Mathematics achievement (academic achievement).

**Figure 3:**
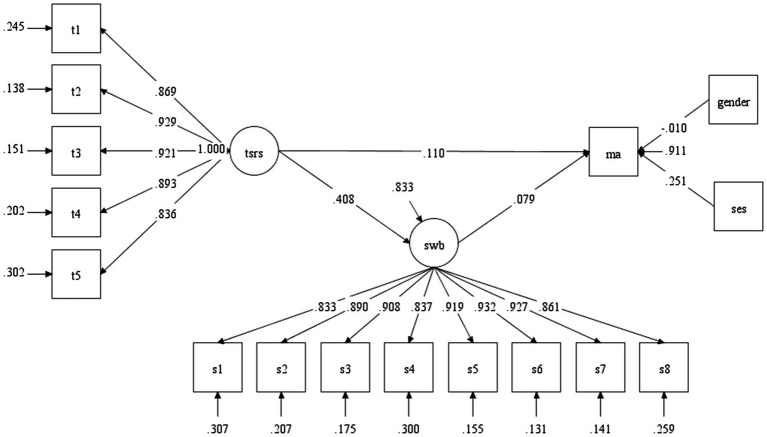
The results of SEM analysis in grade 8. TSR, Teacher-student relationship; SWB,  Subjective well-being; MA ,  Mathematics achievement (academic achievement).

As shown in Figures 2, 3, we found a similar pattern in the relationships among the teacher-student relationship, subjective well-being, and academic achievement. First, the teacher-student relationship has a positive effect on student’s academic achievement in both grades ($\beta_{grade\;4}=0.045$, *p* < 0.001$;\beta_{grade\;8}=0.110$, *p* < 0.001). Second, the teacher-student relationship has a significant influence on students’ subjective well-being in two grade levels ($\beta_{grade\;4}=0.270$, *p* < 0.001; $\beta_{grade\;8}=.408$, *p* < 0.001). Third, subjective well-being has a positive effect on students’ academic achievement for students in grade 4 and grade 8 ($\beta_{grade\;4}=0.123$, *p* < 0.001; $\beta_{grade\;8}=0.079$, *p* < 0.001). In a word, the positive link between the teacher-student relationship and academic achievement was positively mediated by subjective well-being for both graders.

Furthermore, this study also explored the mediating effect of subjective well-being. The bias-corrected bootstrap method was conducted to test the mediating effect of subjective well-being in the two grades (Gootzeit and Markon, 2011; see Table 5). The results showed that the coefficient of the mediation path in each grade was significant (0 was not included in the 95% confidence interval), and the direct effects of teacher-student relationships on mathematics performance in each grade were significant (see Figures 2, 3), suggesting that subjective well-being played a partial mediating role for the fourth graders and the eighth graders. The proportion/percentage of the mediating effects in the total effects were 42.8% (grade 4) and 22.7% (grade 8; Table 6).

**Table 6:** Bias-correlated bootstrap test on mediating effects in grader 4 and grade 8.

Paths	Grade 4			Grade 8		
	Standardized	95% CI		Standardized	95% CI	
		Low	High		Low	High
Total effect	0.077***			0.141***		
TSR→SWB → AA	0.033***	0.029	0.037	0.032***	0.024	0.041

TSR, Teacher-student relationship; SWB, Subjective well-being; AA, Academic Achievement. ***p < 0.001.

## Discussion

The present study extended previous research and provided us more depth understanding with among teacher-student relationship (TSR), subjective well-being (SWB), and academic achievement (AA) in each grade (Year 4 and Year 8). We will discuss the key findings below.

### Performance of fourth and eighth graders in the TSR, SWB, and AA

First, the results found that the TSR of fourth graders was significantly higher than eighth graders, which was generally consistent with previous studies (Hamre and Pianta, 2001; Košir and Tement, 2014; Roorda et al., 2017). Compared to middle school students, elementary school students were more likely to feel the care and love from their teachers under the guidance of attachment theory (Moritz Rudasill et al., 2010; Roorda et al., 2011; Zhou et al., 2020). In addition, pupils in China did not have excessive academic pressure, which has led to much less conflict between teachers and students (Zhou et al., 2021). Conversely, middle school students had more academic pressure and teachers maintained good relationships with those students who perform well, while for underachieving students, the relationships between teachers and them became more delicate (Zhang and Jiang, 2009). This, coupled with the fact that middle school students are in a period of rebellion in which students usually like to go against the teachers (McMahon et al., 2022), may also contribute to the fact that the teacher-student relationship does not perform as well as those of primary school students.

Second, the SWB of fourth graders was also significantly higher than eighth graders, which was in congruence with earlier studies (Yao et al., 2017; Steinmayr et al., 2019). In the Chinese educational context, academic pressure directly caused middle school students to spend more time on the study (Wen et al., 2020). However, students sometimes were not willing to put in more time to study the subjects they may not like, but they must do so, which will naturally make them feel less happy. Compared with elementary school students, they were freer and have less academic pressure (Kim et al., 2016). Recently, the *Ministry of Chinese Education* has also issued a notice *“Strengthening the administration of examinations in compulsory education schools”* that students in the lower elementary school were not required to take final exams instead of using the performance-based assessment method. This may be the main reason for the differences in subjective well-being among primary and secondary school students.

Third, the AA of fourth graders was also significantly higher than eighth graders, which is the same as Chen (2022) study. Chen (2022) explored the difference in mathematics performance between fourth graders and eighth graders based on the TIMSS data. TIMSS test was mainly based on the national curriculum, which is also the same as this study. Specifically, this study was based on *the primary and secondary national mathematics curriculum standards*, and the performance of students in the two grades only reflected how well students were meeting the curriculum standards but were not necessarily comparable.

Based on the discussion, the results not only provide further evidence for China but also expand our in-depth understanding of the relevant issues.

### The structural equation modeling (TSR, SWB, and AA) in each group

According to the results of correlation analysis, we found that SES and gender were significantly correlated with all other variables, so it was very necessary to control the two variables to make the relationships among key variables closer to the truth (Zhou et al., 2022). In addition, the results also showed that TSR was positively correlated with the MA of fourth graders and eighth graders, which was generally consistent with previous studies (Košir and Tement, 2014; Zhou et al., 2020, 2021). Košir and Tement (2014) found that there existed a positive relationship between TSR and students’ academic achievement, which can be explained by the attachment theory and motivation theory Zhou et al. (2020) also found similar results that positive TSR can promote fourth graders’ improvement in mathematics problem-solving. Thus, this result provides a good basis for the subsequent exploration of the structural relationships among TSR, SWB, and AA.

Based on these built theories (attachment theory, self-determination theory, and the theory of broaden-and-build of positive emotions), we further conducted the SEM analysis in each group after controlling for SES and gender. The results illustrated that the model fit indices in each group are both acceptable and TSR can promote students’ academic achievement by the partial mediating role of SWB in each group. In other words, TSR can not only directly improve students’ AA, but can also indirectly improve students’ AA through SWB. This influence mechanism is consistent for both elementary and secondary school students. Compared with previous (Cornelius-White, 2007; Hughes et al., 2008; Roorda et al., 2011), these results further expanded prior studies to show the important role of SWB instead of merely focusing on the direct relationship between TSR and academic achievement. Specifically, subjective well-being as an important affective factor is not only influenced by the external environment but also has an impact on students’ behavioral performance (Diener et al., 2018). As the findings of this study demonstrate, a supportive TSR creates a safe and enjoyable classroom atmosphere in which students feel a sense of well-being and satisfaction with their learning and life (Siedlecki et al., 2014; Bakadorova and Raufelder, 2018), resulting in an intrinsic drive to take the initiative to express their ideas on issues, engage actively in classroom activities, communicate effectively with their peers, and ultimately contribute to their progress in academic achievement (Diener et al., 2018). This explanation brings the three theories mentioned above together in an organic way, working together in education and teaching practice.

### Comparison of direct and indirect effects of SEM model in each group

In this study, we further conducted the test of mediating effect using the bias-corrected bootstrap method to know the significance of direct effect and indirect effect and calculated the percentage of the direct effect and indirect effect to the total effect. The results showed that SWB played a partial mediating role for the fourth graders and the eighth graders, and the rates of mediating effect were 42.8% (Year 4) and 22.7% (Year 8). The result sheds light on the fact that the mediating effect of SWB is larger for fourth-grade students than that for eighth-grade students. However, in previous studies, few researchers compared the importance of SWB at the primary and secondary levels. Most researchers mainly focused on the factors that influence SWB such as social support (Siedlecki et al., 2014; Diener et al., 2018) and the outcomes of SWB such as academic achievement (Ng et al., 2015; Bücker et al., 2018). Therefore, the main contribution of this study is to focus on the grade differences of the mediating effect of SWB.

Why SWB plays a larger mediating role for elementary students? First, from the perspective of cognitive development, pupils’ cognitive development level is immature, they are more influenced by teacher instructions, which makes them more susceptible to changes in their sense of well-being or learning motivation that can affect their academic achievement (Dhuey, 2013; Altermatt, 2016; Wang and Tsai, 2017). However, middle school students, in the puberty stage, have their ideas on issues, so they are less susceptible to teachers’ instructions, which will directly lead to lower academic achievement (Cleary and Chen, 2009; Devlin et al., 2022). In addition, from the perspective of the external conditions, the focus at elementary school is on a typically supportive, master-based orientation, while at middle school, there is a shift to a performance-focused environment characterized by increased expectations of academic productivity, more intensive and teacher-directed teaching, and a greater focus on normative comparisons and high-stakes outcomes (Cleary and Chen, 2009). Therefore, the mediating role of SWB for elementary school students is greater than for middle school students. Based on the role of grade levels, this study provided more exciting results and further extended relative studies.

### Limitations and future research direction

Although this study has been very comprehensive, there are still some limitations. First, we have only focused on a few key factors that influence student achievement, but the factors that influence student achievement are very complex such as teaching methods or classroom management, so in the future, it may be more educationally relevant to develop more complex structural models based on relevant theories to explain them. Second, although mathematics achievement, which is commonly used internationally as a representative of academic achievement, was chosen for this study, the results may be better if future studies consider academic achievement in a wider range of subjects. Third, only fourth and eighth graders were selected as representatives of elementary and middle school students in this study, and although this is the same selection as the TIMSS test, it might have been better to have selected more grades when generalizing.

## Conclusion

This study, which utilized large-scale survey data of Chinese elementary school students and middle school students, mainly explored how TSR promotes students’ AA through the mediating role of SWB in each group. Specifically, this study has made the following contributions: (1) compared the differences of TSR, SWB, and AA for fourth graders and eighth graders, which provided more rich information; (2) justified the applicability of the attachment theory, the self-determination theory, and the theory of broaden-and-build of positive emotions to Chinese participants, indicating the robustness of related theories; (3) extends previous studies by paying more attention to the percentage of direct and indirect effects of two models in each group, which provided more valuable information for elementary teachers and middle school teachers aiming to improve students’ academic performance.

## Data availability statement

The original contributions presented in the study are included in the article/supplementary materials, further inquiries can be directed to the corresponding author.

## Author contributions

DZ: conceptualization, methodology, software, validation, formal analysis, visualization, writing—original draft preparation, writing—review and editing, and funding acquisition. SL: writing—review and editing. HZ: formal analysis. JL: investigation, resources, data curation, and project administration. YM: writing—review and editing, and supervision. All authors contributed to the article and approved the submitted version.

## Funding

This study is supported by “the Fundamental Research Funds for the Central Universities” (Grant number: 135211001).

## Conflict of interest

The authors declare that the research was conducted in the absence of any commercial or financial relationships that could be construed as a potential conflict of interest.

## Publisher’s note

All claims expressed in this article are solely those of the authors and do not necessarily represent those of their affiliated organizations, or those of the publisher, the editors and the reviewers. Any product that may be evaluated in this article, or claim that may be made by its manufacturer, is not guaranteed or endorsed by the publisher.

## References

[ref1] Ai X. (2015). Academic burden of primary and secondary school students: concepts, attribution, and responses [*Chinese*]. J. Southeast University (Social Sciences Edition) 41, 93–97. doi: 10.13718/j.cnki.xdsk.2015.04.012

[ref2] Altermatt E. R. (2016). Grade-level declines in perceived academic support from peers: a moderated mediation analysis. J. Early Adolesc. 37, 760–773. doi: 10.1177/027243161562456

[ref3] Amholt T. T. Dammeyer J. Carteri R. Niclasen J. (2020). Psychological well-being and academic achievement among school-aged children: a systematic review. Child Indic. Res. 13, 1523–1548. doi: 10.1007/s12187-020-09725-9, PMID: 27306431

[ref4] Bakadorova O. Raufelder D. (2018). The essential role of the teacher-student relationship in students’ need satisfaction during adolescence. J. Appl. Dev. Psychol. 58, 57–65. doi: 10.1016/j.appdev.2018.08.004

[ref01] Baker J. A. Grant S. Morlock L. (2008). The teacher–student relationship as a developmental context for children with internalizing or externalizing behavior problems. Sch. Psychol. Q. 23, 3–15. doi: 10.1037/1045-3830.23.1.3

[ref5] Barile J. P. Donohue D. K. Anthony E. R. Baker A. M. Weaver S. R. Henrich C. C. (2012). Teacher-student relationship climate and school outcomes: implications for educational policy initiatives. J. Youth Adol. 41, 256–267. doi: 10.1007/s10964-011-9652-8, PMID: 21404108

[ref6] Bortes C. Ragnarsson S. Strandh M. Petersen S. (2021). The bidirectional relationship between subjective well-being and academic achievement in adolescence. J. Youth Adolesc. 50, 992–1002. doi: 10.1007/s10964-021-01413-3, PMID: 33675505 PMC8043926

[ref7] Bücker S. Nuraydin S. Simonsmeier B. A. Schneider M. Luhmann M. (2018). Subjective well-being and academic achievement: a meta-analysis. J. Res. Pers. 74, 83–94. doi: 10.1016/j.jrp.2018.02.007, PMID: 33870638

[ref8] Campbell A. (1976). Subjective measures of well-being. Am. Psychol. 31, 117–124. doi: 10.1037/0003-066X.31.2.117, PMID: 1267244

[ref9] Chen X. (2022). The effects of individual- and class-level achievement on attitudes towards mathematics: an analysis of Hong Kong students using TIMSS 2019. Stud. Educ. Eval. 72:101113. doi: 10.1016/j.stueduc.2021.101113

[ref10] Chong W. H. Huan V. S. Quek C. L. Yeo L. S. Ang R. P. (2010). Teacher-student relationship: the influence of teacher interpersonal behaviours and perceived beliefs about teachers on the school adjustment of low achieving students in Asian middle schools. Sch. Psychol. Int. 31, 312–328. doi: 10.1177/0143034310366207

[ref11] Cleary T. J. Chen P. P. (2009). Self-regulation, motivation, and math achievement in middle school: variations across grade level and math context. J. Sch. Psychol. 47, 291–314. doi: 10.1016/j.jsp.2009.04.002, PMID: 19712778

[ref12] Cornelius-White J. (2007). Learner-centered teacher-student relationships are effective: a meta-analysis. Rev. Educ. Res. 77, 113–143. doi: 10.3102/003465430298563

[ref13] Cortina J. M. (1993). What is coefficient alpha? An examination of theory and applications. J. Appl. Psychol. 78, 98–104. doi: 10.1037/0021-9010.78.1.98

[ref14] Dalton B. (2012). Grade level and science achievement: US performance in cross-national perspective. Comp. Educ. Rev. 56, 125–154. doi: 10.1086/660745

[ref15] Datu J. A. D. King R. B. (2018). Subjective well-being is reciprocally associated with academic engagement: a two-wave longitudinal study. J. Sch. Psychol. 69, 100–110. doi: 10.1016/j.jsp.2018.05.007 30558746

[ref16] Davis H. A. (2006). Exploring the contexts of relationship quality between middle school students and teachers. Elem. Sch. J. 106, 193–223. doi: 10.1086/501483

[ref17] Devlin B. L. Jordan N. C. Klein A. (2022). Predicting mathematics achievement from subdomains of early number competence: differences by grade and achievement level. J. Exp. Child Psychol. 217:5354. doi: 10.1016/j.jecp.2021.105354, PMID: 35078085

[ref18] Dhuey E. (2013). Middle school or junior high? How grade-level configurations affect academic achievement. Can. J. Econ. 46, 469–496. doi: 10.1111/caje.12020, PMID: 36641673

[ref19] Diener E. Emmons R. A. (1984). The independence of positive and negative affect. J. Pers. Soc. Psychol. 47, 1105–1117. doi: 10.1037/0022-3514.47.5.1105 6520704

[ref20] Diener E. Oishi S. Tay L. (2018). Advances in subjective well-being research. Nat. Hum. Behav. 2, 253–260. doi: 10.1038/s41562-018-0307-6, PMID: 30936533

[ref21] Diener E. Ryan K. (2009). Subjective well-being: a general overview. S. Afr. J. Psychol. 39, 391–406. doi: 10.1177/008124630903900402, PMID: 36615518

[ref22] Diener E. Suh E. M. Lucas R. E. Smith H. L. (1999). Subjective well-being: three decades of progress. Psychol. Bull. 125, 276–302. doi: 10.1037/0033-2909.125.2.276, PMID: 36502170

[ref23] Fredrickson B. L. (2001). The role of positive emotions in positive psychology: the broaden-and-build theory of positive emotions. Am. Psychol. 56, 218–226. doi: 10.1037/0003-066X.56.3.218, PMID: 11315248 PMC3122271

[ref24] Frymier A. B. Houser M. L. (2000). The teacher student relationship as an interpersonal relationship. Commun. Educ. 49, 207–219. doi: 10.1080/03634520009379209, PMID: 36620252

[ref25] Funder D. C. Ozer D. J. (2019). Evaluating effect size in psychological research: sense and nonsense. Adv. Methods Pract. Psychol. Sci. 2, 156–168. doi: 10.1177/2515245919847202

[ref26] Gagne M. Deci E. (2005). Self-determination theory and work motivation. J. Organ. Behav. 26, 331–362. doi: 10.1002/job.322, PMID: 36626154

[ref27] Gootzeit J. Markon K. (2011). Factors of PTSD: differential specificity and external correlates. Clin. Psychol. Rev. 31, 993–1003. doi: 10.1016/j.cpr.2011.06.005, PMID: 21741913

[ref28] Hamre B. K. Pianta R. C. (2001). Early teacher-child relationships and the trajectory of children’s school outcomes through eighth grade. Child Dev. 72, 625–638. doi: 10.1111/1467-8624.00301, PMID: 11333089

[ref29] Hu L. T. Bentler P. M. (1999). Cutoff criteria for fit indexes in covariance structure analysis: conventional criteria versus new alternatives. Struct. Equ. Model. 6, 1–55. doi: 10.1080/10705519909540118

[ref30] Hu P. Zhang J. (2017). A pathway to learner autonomy: a self-determination theory perspective. Asia Pac. Educ. Rev. 18, 147–157. doi: 10.1007/s12564-016-9468-z

[ref31] Hughes J. N. Luo W. Kwok O. Loyd L. (2008). Teacher-student support, effortful engagement, and achievement: a 3-year longitudinal study. J. Educ. Psychol. 100, 1–14. doi: 10.1037/0022-0663.100.1.1, PMID: 19578558 PMC2705122

[ref32] Johnson R. B. Christensen L. B. (2019). Educational research: Quantitative, qualitative, and mixed approaches. Los Angeles: United States: SAGE.

[ref33] Kim Y. Kwak K. Lee S. (2016). Does optimism moderate parental achievement pressure and academic stress in Korean children? Curr. Psychol. 35, 39–43. doi: 10.1007/s12144-015-9355-5

[ref34] King R. B. McInerney D. M. Ganotice F. A. Jr. Villarosa J. (2015). Positive affect catalyzes academic engagement: cross-sectional, longitudinal, and experimental evidence. Learn. Individ. Differ. 39, 64–72. doi: 10.1016/j.lindif.2015.03.005

[ref35] Koca F. (2016). Motivation to learn and teacher-student relationship. J. Int. Educ. Leader. 6, 1–20.

[ref36] Košir K. Tement S. (2014). Teacher-student relationship and academic achievement: a cross-lagged longitudinal study on three different age groups. Eur. J. Psychol. Educ. 29, 409–428. doi: 10.1007/s10212-013-0205-2

[ref37] Lei H. Wang X. Chiu M. M. Du M. Xie T. (2022). Teacher-student relationship and academic achievement in China: evidence from a three-level meta-analysis. Sch. Psychol. Int. doi: 10.1177/01430343221122453

[ref38] Li R. (2021). The role of teacher-student interpersonal relations in flipped learning on student engagement. Front. Psychol. 12:741810. doi: 10.3389/fpsyg.2021.741810, PMID: 34456838 PMC8387675

[ref39] Liu Q. Du X. Zhao S. Liu J. Cai J. (2019). The role of memorization in students’ self-reported mathematics learning: a large-scale study of Chinese eighth-grade students. Asia Pac. Educ. Rev. 20, 361–374. doi: 10.1007/s12564-019-09576-2

[ref40] Liu J. Peng P. Luo L. (2020). The relation between family socioeconomic status and academic achievement in China: a meta-analysis. Educ. Psychol. Rev. 32, 49–76. doi: 10.1007/s10648-019-09494-0

[ref41] Luhmann M. (2017). Using big data to study subjective well-being. Current Option Behav. Sci. 18, 28–33.

[ref42] Ma Z. (2015). The relationship between primary and secondary school students' learning ability, learning environment and academic achievement: based on the survey and analysis of 13,477 primary and secondary school students [Chinese]. J. Chin. Soc. Educ. 8, 48–60.

[ref43] McInerney D. M. Cheng R. W. Y. Mok M. M. C. Lam A. K. H. (2012). Academic self-concept and learning strategies direction of effect on student academic achievement. J. Advanced Acad. 23, 249–269. doi: 10.1177/1932202X12451020, PMID: 31901646

[ref44] McMahon S. D. Cafaro C. L. Bare K. Zinter K. E. Murillo Y. G. Subotnik R. (2022). Rates and types of student aggression against teachers: a comparative analysis of U.S. elementary, middle, and high schools. Soc. Psychol. Educ. 25, 767–792. doi: 10.1007/s11218-022-09706-6

[ref45] Mikulincer M. Shaver P. R. (2010). Attachment in adulthood: Structure, dynamics, and change. Guilford Press, New York.

[ref46] Mikulincer M. Shaver P. R. Pereg D. (2003). Attachment theory and affect regulation: the dynamics, development, and cognitive consequences of attachment-related strategies. Motiv. Emot. 27, 77–102. doi: 10.1023/A:1024515519160

[ref47] Moritz Rudasill K. Reio T. G. Jr. Stipanovic N. Taylor J. E. (2010). A longitudinal study of student-teacher relationship quality, difficult temperament, and risky behavior from childhood to early adolescence. J. Sch. Psychol. 48, 389–412. doi: 10.1016/j.jsp.2010.05.001, PMID: 20728689

[ref48] Ng Z. J. Huebner S. E. Hills K. J. (2015). Life satisfaction and academic performance in early adolescents: evidence for reciprocal association. J. Sch. Psychol. 53, 479–491. doi: 10.1016/j.jsp.2015.09.004, PMID: 26563600

[ref49] Nowell A. Hedges L. V. (2018). Trends in gender differences in academic achievement from 1960 to 1994: an analysis of differences in mean, variance, and extreme scores. Sex Roles 39, 21–43. doi: 10.1023/A:1018873615316

[ref50] Organisation for Economic Co-Operation and Development [OECD]. (2017). PISA 2015 technical report. Retrieved from http://www.oecd.org/pisa/sitedocument/PISA-2015-technical-report-final.pdf.

[ref51] Pekrun R. (2006). The control-value theory of achievement emotions: assumptions, corollaries, and implications for educational research and practice. Educ. Psychol. Rev. 18, 315–341. doi: 10.1007/s10648-006-9029-9, PMID: 22364457

[ref52] Poling D. V. Van Loan C. L. Garwood J. D. Zhang S. Riddle D. (2022). Enhancing teacher-student relationship quality: a narrative review of school-based interventions. Educ. Res. Rev. 37:100459. doi: 10.1016/j.edurev.2022.100459

[ref53] Quin D. (2017). Longitudinal and contextual associations between teacher–student relationships and student engagement: a systematic review. Rev. Educ. Res. 87, 345–387. doi: 10.3102/0034654316669434

[ref54] Robinson C. D. (2022). A framework for motivating teacher-student relationships. Educ. Psychol. Rev. 34, 2061–2094. doi: 10.1007/s10648-022-09706-0, PMID: 36120964

[ref55] Romero C. Master A. Paunesku D. Dweck C. S. Gross J. J. (2014). Academic and emotional functioning in middle school: the role of implicit theories. Emotion 14, 227–234. doi: 10.1037/a0035490, PMID: 24512251

[ref56] Roorda D. L. Jak S. Zee M. Oort F. J. Koomen H. M. Y. (2017). Affective teacher–student relationships and students’ engagement and achievement: a meta-analytic update and test of the mediating role of engagement. Sch. Psychol. Rev. 46, 239–261. doi: 10.17105/SPR-2017-0035.V46-3

[ref57] Roorda D. L. Koomen H. M. Spilt J. L. Oort F. J. (2011). The influence of affective teacher-student relationships on students’ school engagement and achievement: a meta-analytic approach. Rev. Educ. Res. 81, 493–529. doi: 10.3102/0034654311421793

[ref58] Ryan R. M. Deci E. L. (2006). Self-regulation and the problem of human autonomy: does psychology need choice, self-determination, and will? J. Pers. 74, 1557–1585. doi: 10.1111/j.1467-6494.2006.00420.x, PMID: 17083658

[ref59] Ryan R. M. Deci E. L. (2011). “A self-determination theory perspective on social, institutional, cultural, and economic supports for autonomy and their importance for well-being” in Human autonomy in cross-cultural advancements in positive psychology. eds. Chirkov V. I. Ryan R. M. Sheldon K. M., vol. 1 (New York: Springer science business media), 45–64.

[ref60] Ryan R. M. Deci E. L. (2017). Self-determination theory. Basic psychological needs in motivation, development, and wellness. New York, NY: Guilford Press.

[ref61] Selvitopu A. Kaya M. (2021). A meta-analytic review of the effect of socioeconomic status on academic performance. J. Educ. 12:978. doi: 10.1177/00220574211031978

[ref62] Siedlecki K. L. Salthouse T. A. Oishi S. Jeswani S. (2014). The relationship between social support and subjective well-being across age. Soc. Indic. Res. 117, 561–576. doi: 10.1007/s11205-013-0361-4, PMID: 25045200 PMC4102493

[ref63] Sirin S. R. (2005). Socioeconomic status and academic achievement: a meta-analytic review of research. Rev. Educ. Res. 75, 417–453. doi: 10.3102/00346543075003417

[ref64] Spilt J. L. Koomen H. M. Y. Thijs J. T. (2011). Teacher wellbeing: the importance of teacher-student relationships. Educ. Psychol. Rev. 23, 457–477. doi: 10.1007/s10648-011-9170-y, PMID: 36275306

[ref65] Steinmayr R. Weidinger A. F. Schwinger M. Spinath B. (2019). The importance of students’ motivation for their academic achievement: replicating and extending previous findings. Front. Psychol. 10:1730. doi: 10.3389/fpsyg.2019.01730 31417459 PMC6685139

[ref66] Tay L. Diener E. (2011). Needs and subjective well-being. J. Pers. Soc. Psychol. 101, 354–365. doi: 10.1037/a0023779, PMID: 21688922

[ref67] Van Petegem K. Aelterman A. Van Keer H. Rosseel Y. (2007). The influence of student characteristics and interpersonal teacher behaviour in the classroom on student’s wellbeing. Soc. Indic. Res. 85, 279–291. doi: 10.1007/s11205-007-9093-7, PMID: 20014644

[ref68] Wang Y. L. Tsai C. C. (2017). Grade level differences in high school students’ conceptions of and motives for learning science. Res. Sci. Educ. 49, 1213–1229. doi: 10.1007/s11165-017-9651-1

[ref69] Wang J. Wang X. (2020). Structural equating modeling: Applications using Mplus. United States: John Wiley & Sons Ltd.

[ref70] Wen X. Lin Y. Liu Y. Starcevich K. Yuan F. Yuan Z. (2020). A latent profile analysis of anxiety among junior high school students in less developed rural regions of China. Int. J. Environ. Res. Public Health 17:4079. doi: 10.3390/ijerph17114079, PMID: 32521646 PMC7312008

[ref71] Yao Y. Kong Q. Cai J. (2017). Investigating elementary and middle school students’ subjective well-being and mathematical performance in Shanghai. Int. J. Sci. Math. Educ. 16, 107–127. doi: 10.1007/s10763-017-9827-1

[ref72] Zhang X. He L. Zheng X. (2004). Adolescent students’ life satisfaction: its construct and scale development. Psychol. Sci. 27, 1257–1260. doi: 10.16719/j.cnki.1671-6981.2004.05.068

[ref73] Zhang B. Jiang T. (2009). A correlation analysis of teacher-student relationship and academic achievement in junior high schools. Psychol. Sci. 32, 1015–1017. doi: 10.16719/j.cnki.1671-6981.2009.04.068

[ref74] Zhou D. Du X. F. Hau K. T. Luo H. F. Feng P. T. Liu J. (2020). Teacher-student relationship and mathematical problem-solving ability: mediating roles of self-efficacy and mathematical anxiety. Educ. Psychol. 40, 473–489. doi: 10.1080/01443410.2019.1696947

[ref75] Zhou D. Liu J. Liu J. (2021). On the different effects of teacher-student rapport on urban and rural students’ math learning in China: an empirical study. Psychol. Sch. 58, 268–285. doi: 10.1002/pits.22446

[ref76] Zhou D. Liu J. Wang T. Liu J. Li G. (2022). Relationships among problematic smartphone use, mathematics anxiety, learning interest, and achievement: a multiple mediation model. Comput. Hum. Behav. 129:7171. doi: 10.1016/j.chb.2021.107171

